# Comparative Epidemiology and Resistance Trends of Common Urinary Pathogens in a Tertiary-Care Hospital: A 10-Year Surveillance Study

**DOI:** 10.3390/medicina55070356

**Published:** 2019-07-09

**Authors:** Márió Gajdács, Marianna Ábrók, Andrea Lázár, Katalin Burián

**Affiliations:** 1 Department of Pharmacodynamics and Biopharmacy, Faculty of Pharmacy, University of Szeged, Eötvös utca 6., 6720 Szeged, Hungary; 2 Institute of Clinical Microbiology, Faculty of Medicine, University of Szeged, Semmelweis utca 6., 6725 Szeged, Hungary; 3 Department of Medical Microbiology and Immunobiology, Faculty of Medicine, University of Szeged, Dóm tér 10., 6720 Szeged, Hungary

**Keywords:** urinary tract infection, infectious disease, antibiotic, resistance, indicator, epidemiology, fosfomycin, ESBL, *Escherichia coli*, *Klebsiella*

## Abstract

*Background and Objective:* Urinary tract infections (UTIs) are common in human medicine, affecting large patient populations worldwide. The principal cause of UTIs is uropathogenic *Escherichia coli* (UPEC) and *Klebsiella*, both in community and nosocomial settings. The assessment of local data on prevalence and resistance is essential to evaluate trends over time and to reflect on the national situation, compared to international data, using the methods of analytical epidemiology. *Materials and Methods:* The aim of this study was to assess resistance trends and epidemiology of UTIs caused by *E. coli* and *Klebsiella* species in inpatients and outpatients at a tertiary-care hospital in Hungary, using microbiological data. To evaluate resistance trends, several antibiotics were chosen as indicator drugs, based on local utilization data. *Results:* *E. coli* was the most prevalent isolate, representing 56.75 ± 4.86% for outpatients and 42.29 ± 2.94% for inpatients. For *E. coli*, the ratio of resistant strains for several antibiotics was significantly higher in the inpatient group, while in *Klebsiella*, similar trends were only observed for gentamicin. Extended-spectrum β-lactamase (ESBL)-producing isolates were detected in 4.33–9.15% and 23.22–34.22% from outpatient, 8.85–38.97% and 10.89–36.06% from inpatient samples for *E. coli* and *Klebsiella*, respectively. *Conclusions:* Resistance developments in common UTI pathogens (especially to fosfomycin, sulfamethoxazole-trimethoprim, fluoroquinolones, and 3rd generation cephalosporins), seriously curb therapeutic options, especially in outpatient settings.

## 1. Introduction

Urinary tract infections (UTIs) are some of the most common infections in human medicine, affecting a large patient population (around 150 million cases/year) to various extents, irrespective of age and gender [1,2]. Both community-acquired (representing 10–30% of infections) and nosocomial UTIs (accounting for 25–50% of infections) should be considered as an important factor of morbidity (they are often associated with complications, sequelae, recurrence and decreased quality of life), a serious public health issue and an economic burden (the therapy and care of these patients alone is estimated to cost around 5 billion US$) [3,4,5]. The causative agents of UTIs are diverse, especially in nosocomial settings (where prolonged catheterization and immunosuppression facilitates the occurrence of non-conventional urinary pathogens) [6,7].

However, the most common causative agents of UTIs are the members of the *Enterobacteriaceae* family (or the *Enterobacterales* order, based on recent taxonomic changes [8]), followed by (in decreasing order of frequency) some Gram-positive bacteria (*Enterococcus faecalis*, Group B streptococci, *Staphylococcus saprophyticus* and *S. aureus*), atypical microorganisms (*Mycoplasma*, *Ureaplasma* species), non-fermenting Gram-negative bacteria (*Pseudomonas*, *Acinetobacter*) and *Candida* spp. [1,3,4,5,6,9,10]. The principal cause of UTIs (>80%) are uropathogenic *Escherichia coli* (UPEC) and *Klebsiella* species (more specifically, *K. pneumoniae* and *K. oxytoca*), both in the community and nosocomial settings. *E. coli* is described as the etiological agent in 60–90% of urinary tract infections, while *Klebsiella* species accounts for 3–20% of cases [3,11,12,13,14,15]. These species are successful pathogens in the urinary system, as they possess the relevant virulence factors required to successfully survive on and adhere to urinary epithelium, cause tissue damage and to ascend to the upper urinary tract (leading to complicated UTIs) [16,17,18,19,20,21]. These virulence factors include the lipopolysaccharide (LPS), polysaccharide capsule, outer membrane vesicles, iron-uptake (aerobactin) and siderophore receptors, adhesins, Type-1 fimbriae, cytotoxins and urease production. In fact, UPEC may be classified into several phylogroups (namely A, B1, B2 and D) based on the presence of pathogenicity islands (PAI) and virulence factor expression [16,17,18,19,20,21]. The relevance of *Klebsiella* species has been described in nosocomial UTIs, as they are ubiquitous in the hospital environment and are able to survive on both living (e.g., patient’s skin) and abiotic (e.g., wards, catheters) surfaces [22,23,24].

The recommended drugs for the (empiric) treatment of uncomplicated urinary tract infections include nitrofurantoin, fosfomycin and sulfamethoxazole/trimethoprim (if local resistance levels do not exceed 20%), which are all available *per os*; in case of resistance or hypersensitivity to these drugs, or if complicated UTIs (e.g., pyelonephritis) need to be treated, other therapeutic options, such as β-lactam antibiotics (third generation cephalosporins, e.g., ceftriaxone; carbapenems), fluoroquinolones and aminoglycosides should also be considered [3,5,25,26]. The group of β-lactam antibiotics is especially important, because in several vulnerable patient populations (children, pregnant women, patients with liver/kidney failure), there drugs are the first-choice agents, due to the debilitating side effect-profile of the alternate drugs [27]. Extended-spectrum β-lactamases (ESBLs) are plasmid-encoded enzymes, capable of hydrolyzing penicillin-derivatives and cephalosporins (including third and fourth generation cephalosporins); in contrast, AmpC-type β-lactamases are mostly chromosomally-encoded (although they have also been described on plasmids), capable of hydrolyzing penicillin-derivatives and cephalosporins (including third generation cephalosporins and aztreonam, but not fourth generation cephalosporins). ESBLs are mainly Ambler Class A enzymes, that are inhibited by “first-generation” β-lactamase-inhibitors (such as clavulanic acid or sulbactam), while AmpC enzymes are Class C enzymes that are not inhibited by these adjuvants [28,29,30,31,32,33]. Among *Enterobacteriaceae*, the prevalence of ESBLs and plasmid-encoded AmpCs are the highest in *Klebsiella* spp. and *E. coli* [34]. Additionally, *Klebsiella* species have shown to be great vectors for these plasmids and they provide a suitable genetic environment to mutations [28,29,30,31,32,33]. Since the 2000s, *bla*_CTX-M_-type ESBLs are the most prevalent around the globe, while *bla*_TEM_ and *bla*_SHV_-type enzymes have become less relevant over time [28,29,30,31,32,33,35,36]. Due to their transmissibility, ESBLs are considered a serious public health/infection control issue, especially because these enzymes are encoded on larger-sized plasmids, which may additionally include resistance determinants for aminoglycosides and/or fluoroquinolones [28,37]. ESBL-producers have been associated with increased morbidity and mortality, particularly in intensive care units and in severely immunocompromised patients [38,39,40]. In UTIs with an ESBL-producing isolate (coupled with other resistance mechanisms), carbapenems essentially remain the only safe drug choice, while other “last resort” agents (e.g., tigecycline, colistin, ceftazidime-avibactam) are rarely used, due to their price, pharmacokinetic profile or side effects. Multidrug resistance (MDR) in Gram-negative bacteria is a growing concern, and the treatment of UTIs is increasingly complex challenge for clinicians [25,41,42,43,44]. These uropathogens may be resistant to a wide range of available drugs, necessitating the use of drugs that are only available in intravenous formulations, limiting the care of these patients to inpatient setting or outpatient parenteral antimicrobial therapy (OPAT), if available [45,46].

Various national- and international-level surveillance programs are evaluating and publishing the resistance trends of various Gram-positive and Gram-negative bacteria [6,9,14,47,48,49,50,51,52]. Nevertheless, the epidemiology and antibiotic-susceptibility patterns of urinary tract pathogens vary greatly by region therefore, the assessment of local data is essential to evaluate trends over time and to reflect on the national situation, compared to international data, using the methods of analytical epidemiology [6,9,14,47,48,49,50,51,52]. Additionally, knowledge of the relevant antibiotic-susceptibility patterns of the major bacterial pathogens for UTIs is of utmost importance to allow for the optimal choice for antibiotic therapy [53,54]. The aim of this study was to assess and compare the resistance trends and epidemiology of *E. coli* and *Klebsiella* species in inpatients and outpatients at the Albert Szent-Györgyi Clinical Center (Szeged, Hungary) retrospectively, during a 10-year study period.

## 2. Materials and Methods

### 2.1. Study Design, Data Collection

**Figure 1:**
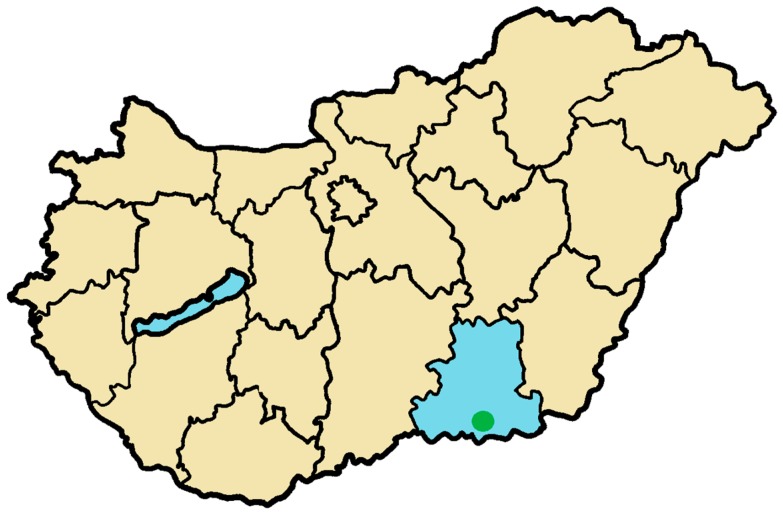
Study site in Hungary (Southern Great Plain of Hungary: in blue; Albert Szent-Györgyi Clinical Center, Szeged: in green).

This retrospective study was carried out using microbiological data collected from the period between the 1st of January 2008 and 31st of December 2017 at the Institute of Clinical Microbiology (University of Szeged), which is the affiliated diagnostic microbiology laboratory of the Albert Szent-Györgyi Clinical Center, a primary- and tertiary-care teaching hospital in the Southern Great Plain of Hungary. The Clinical Center has a bed capacity of 1820-beds (1465 active and 355 chronic beds, respectively) and annually serves more than 400,000 patients in the region, according to the data of the Hungarian National Health Insurance Fund (NEAK), including GP-level care, all the way to specialized medical interventions (Figure 1) [55]. Electronic search in the records of the MedBakter laboratory information system (LIS) for urine samples positive for *E. coli* and *Klebsiella* species was conducted by the authors (M.G., Á.M. and A.L.).

Samples with clinically significant colony counts for the abovementioned bacteria (10^5^ < colony forming units [CFU]/mL; however, this was subject to interpretation, based on the information provided on the request forms for microbiological analysis and relevant international guidelines, e.g., presence of underlying conditions in the genitourinary tract) were included in the data analysis. Only the first isolate per patient was included in the study, however, isolates with different antibiotic-susceptibility patterns were considered as different individual isolates. In addition, patient data was also collected, that were limited to demographic characteristics (age and sex). The study was deemed exempt from ethics review by the Institutional Review Board, and informed consent was not required as data anonymity was maintained.

### 2.2. Identification of Isolates

10 µL of each un-centrifuged urine sample was cultured on UriSelect chromogenic agar plates (Bio-Rad, Berkeley, CA, USA) with a calibrated loop, according to the manufacturer’s instructions and incubated at 37 °C for 24–48 h, aerobically. If the relevant pathogens presented in significant colony count, the plates were passed on for further processing. Between 2008–2012, presumptive phenotypic (biochemical reaction-based) methods and VITEK 2 ID (bioMérieux, Marcy-l’Étoile, France) were used for bacterial identification, while after 2013, this was complemented by matrix-assisted laser desorption/ionization time-of-flight mass spectrometry (MALDI-TOF MS; Bruker Daltonik Gmbh. Gr., Bremen, Germany). The methodology of sample preparation for MALDI-TOF MS measurements was described elsewhere [56,57,58]. Mass spectrometry was performed by the Microflex MALDI Biotyper (Bruker Daltonics, Bremen, Germany) in positive linear mode across the m/z range of 2 to 20 kDa; for each spectrum, 240 laser shots at 60 Hz in groups of 40 shots per sampling area were collected. The MALDI Biotyper RTC 3.1 software (Bruker Daltonics) and the MALDI Biotyper Library 3.1 were used for spectrum analysis.

### 2.3. Antimicrobial Susceptibility Testing

Antimicrobial susceptibility testing (AST) was performed using the Kirby–Bauer disk diffusion method and when appropriate, E-test (Liofilchem, Abruzzo, Italy) on Mueller–Hinton agar (MHA) plates. In addition, for the verification of discrepant results, VITEK 2 AST (bioMérieux, Marcy-l’Étoile, France) was also used. The interpretation of the results was based on EUCAST breakpoints. *Staphylococcus aureus* ATCC 29213, *Enterococcus faecalis* ATCC 29212, *Proteus mirabilis* ATCC 35659, *Escherichia coli* ATCC 25922, *Klebsiella pneumoniae* ATCC 700603 and *Pseudomonas aeruginosa* ATCC 27853 were used as quality control strains.

To evaluate the resistance trends of isolated strains, ciprofloxacin (CIP), ceftriaxone (CRO), meropenem (MER), gentamicin (GEN), sulfamethoxazole/trimetoprim (SXT) and nitrofurantion (NIT; relevant in case of *E. coli*) were chosen as indicator antibiotics, based on local antibiotic utilization data [59,60]. In addition, susceptibility data for fosfomycin (FOS) was also available for the second half (2013–2017) of the study period. FOS susceptibility testing was not routinely performed, only per request of the clinicians or in cases of extensive drug resistance. During data analysis, intermediately-susceptible results were grouped with and reported as resistant.

If extended-spectrum beta-lactamase (ESBL)-production was suspected, detection was carried out based on EUCAST recommendations; since 2011, using AmpC-ESBL Detection Set (MAST Diagnostica GmbH, Reinfeld, Germany) and VITEK 2 AST (bioMérieux, Marcy-l’Étoile, France), according to the manufacturer’s instructions. Carbapenemase-production was suspected in case of reduced susceptibility or resistance to MER, these isolates were sent to a reference laboratory for further processing.

### 2.4. Statistical Analysis

Descriptive statistical analysis (including means or medians with ranges and percentages to characterize data) was performed using Microsoft Excel 2013 (Redmond, WA, USA, Microsoft Corp.). Statistical analyses were performed with SPSS software version 24 (IBM SPSS Statistics for Windows 24.0, Armonk, NY, USA, IBM Corp.), using the χ^2^-test, Student’s t-test and Mann–Whitney U test. The normality of variables was tested using Shapiro–Wilk tests. *p* values < 0.05 were considered statistically significant.

## 3. Results

### 3.1. Demographic Characteristics, Sample Types

**Figure 2:**
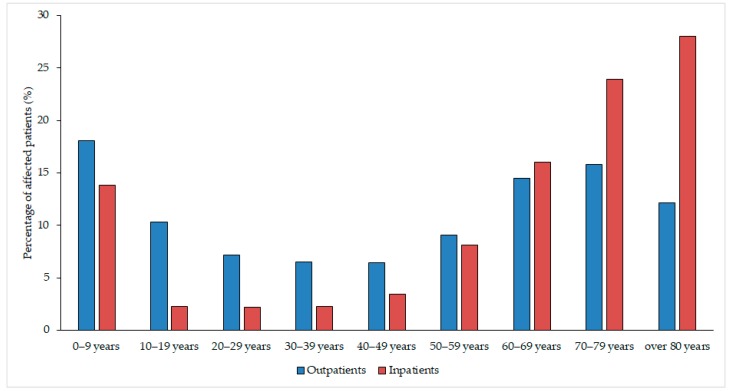
Age distribution of the affected patients in the outpatient and inpatient groups.

The median age of affected patients was 52 years (range: 0.3–97) in the outpatient group with a female-to-male ratio of 3.62 (78.34% female), while in the inpatient group, these values were 71 years (range: 0.4–96) and 2.42 (70.75% female), respectively. The detailed age distribution of patients in both affected patient groups is presented in Figure 2. The difference in the age distribution of the two patient groups was statistically significant (*p* < 0.0001). Among the affected patients, the age groups under 10 years of age (outpatients: 18.04%, inpatients: 13.80%) and over 60 years of age (outpatients: 42.44%, outpatients: 68.04%) were the most numerous. All (100%) samples received from outpatient clinics were voided (midstream) urine, while the sample distribution from the inpatient departments was the following: midstream urine (46.72%) catheter-specimen urine (45.56%), first-stream urine (7.41%) and samples obtained through suprapubic bladder aspiration (0.31%).

### 3.2. Epidemiology of Escherichia coli and Klebsiella spp. Isolates

**Figure 3:**
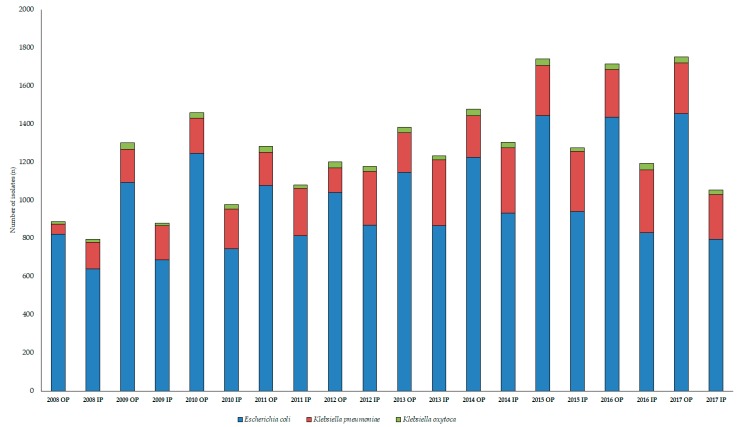
Frequency and species distribution of relevant isolates in inpatient and outpatient samples (2008–2017); IP: inpatient; OP: outpatient.

During the 10-year study period (1 January 2008–31 December 2017), the Institute of Clinical Microbiology received 21,150 urine samples from outpatient clinics and 19,325 samples from inpatient departments that turned out to be positive for a significant urinary pathogen. Out of the positive urine samples, *E. coli* represented the overwhelming majority of all positive urine samples; 56.75 ± 4.86% (range: 46.83–65.98%, lowest in 2008, highest in 2010) for outpatients, while 42.29 ± 2.94% (range: 37.19–45.73%, lowest in 2015, highest in 2010) for inpatients. *K. pneumoniae* was isolated in 8.96 ± 2.29% (range: 2.97–10.37%, lowest in 2012, highest in 2011) for outpatients, while 13.41 ± 2.20% (range: 9.54–15.25%, lowest in 2008, highest in 2011) for inpatient isolates; the isolation rate for *K. oxytoca* was 1.44 ± 0.34% (range: 0.74–1.77%, lowest in 2008, highest in 2011) for outpatients, and 1.16 ± 0.22% (range: 0.74–1.77%, lowest in 2008, highest in 2011) for inpatients. There was significant difference in the isolation frequency of *E. coli* (*p* = 0.0002) and *K. pneumoniae* (*p* = 0.0003) when comparing inpatient and outpatient isolates, while only a numerical tendency could be observed for *K. oxytoca* (*p* > 0.05). The epidemiology and total species distribution of outpatient and inpatient samples is presented in Figure 3.

### 3.3. Antibiotic-Susceptibility Trends among Isolates

**Figure 4:**
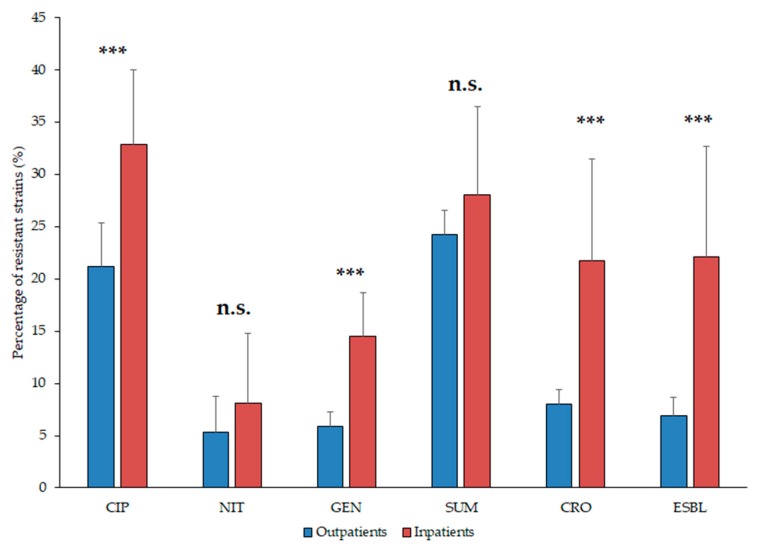
Resistance levels of *Escherichia coli* isolates from inpatient and outpatient urinary tract infections, expressed in the percentage (%) of resistant isolates. Asterisk (***): *p* < 0.001; n.s.: not significant; CIP: ciprofloxacin; NIT: nitrofurantoin; GEN: gentamicin; SUM: sulfamethoxazole/trimethoprim; CRO: ceftriaxone; ESBL: extended-spectrum β-lactamase-producing isolates.

**Figure 5:**
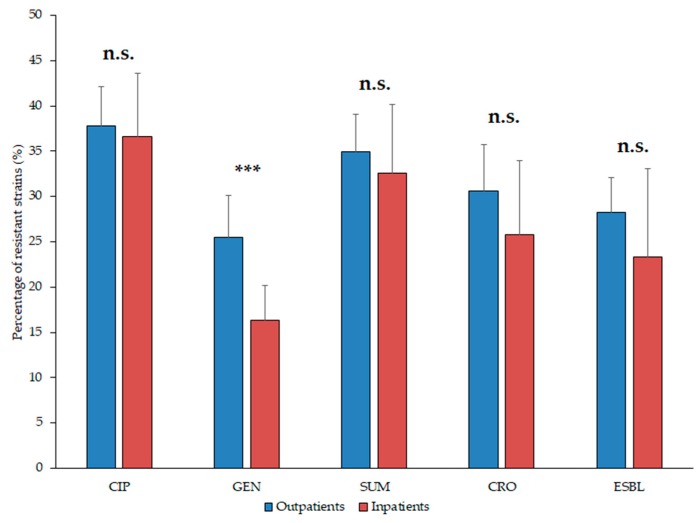
Resistance levels of *Klebsiella* spp. isolates from inpatient and outpatient urinary tract infections, expressed in the percentage (%) of resistant isolates. Asterisk (***): *p* < 0.001; n.s.: not significant; CIP: ciprofloxacin; NIT: nitrofurantoin; GEN: gentamicin; SUM: sulfamethoxazole/trimethoprim; CRO: ceftriaxone; ESBL: extended-spectrum β-lactamase-producing isolates.

The resistance trends of the isolates *E. coli* and *Klebsiella* spp. against ciprofloxacin (CIP), ceftriaxone (CRO), gentamicin (GEN), sulfamethoxazole-trimethoprim (SXT), nitrofurantoin (NIT, relevant in case of *E. coli*) and the ratio of ESBL-producers (relevant since 2011) during the 10-year surveillance period are presented in Table 1 and Table 2, and Figure 4 (*E. coli*) and Figure 5. (*Klebsiella* spp.). Overall, the highest levels of resistance in *E. coli* and *Klebsiella* spp. were detected against CIP and SUM. The ratio of resistant *E. coli* strains in the inpatient group were significantly higher to CIP, GEN and CRO (*p* = 0.0003, *p* < 0.0001 and *p* = 0.0003, respectively), but not in case of NIT and FOS (*p* > 0.05; Figure 4). In addition, *E. coli* resistance levels to the indicator antibiotics were significantly higher (*p* < 0.05) in the second half (2013–2017) of the study period in case of every drug (apart from CRO resistance in the outpatient samples and NIT, where resistance levels were significantly higher in the first five-year-period; Table 1; Figure 4).

For the statistical analysis of resistance trends, *K. pneumoniae* and *K. oxytoca* isolates were grouped together as *Klebsiella* spp., as *K. oxytoca* represented only a minority (~1–1.5%) of isolates; in addition, after a preliminary analysis of the resistance trends among these two species, no differences (statistical or otherwise) could be observed. For *Klebsiella* spp., ratio of resistant strains in the inpatient group were significantly higher to GEN only (*p* = 0.0002; Figure 5). Resistance levels to the indicator antibiotics in *Klebsiella* spp. of inpatient origin were significantly higher (*p* < 0.05) in the second half (2013–2017) of the study period in case of every drug, while no similar trend was observed for the outpatient samples (Table 2). Regarding the outpatient isolates, the resistance levels of *Klebsiella* spp. were significantly higher, compared to *E. coli* to all antibiotics, except for SXT (CIP: *p* < 0.0001; GEN: *p* < 0.0001; CRO *p* = 0.0003), while during the analysis of inpatient samples, no similar trends were observed (*p* > 0.05).

ESBL-positivity (or simultaneous ESBL and AmpC-positivity) was detected in 6.94 ± 1.76% of outpatient and 22.09 ± 10.57% of inpatient *E. coli* isolates (*p* = 0.0028); in *Klebsiella* spp., these ratios were 28.23 ± 3.87% for outpatient and 23.36 ± 9.73% for inpatient isolates (*p* = 0.243). As of 2013, FOS susceptibility testing was performed for 15.63% of *E. coli* and 16.34% of *Klebsiella* spp. isolates in the inpatient group, while for outpatient isolates, 5.67% of *E. coli* and 17.77% of *Klebsiella* spp. were tested. Regarding inpatient samples, resistance to FOS was present in 14.66% of *E. coli* and 23.47% of *Klebsiella* spp., while in outpatient samples, this was 8.89% for *E. coli* (*p* = 0.0035) and 15.92% for *Klebsiella* spp. (*p* = 0.047), respectively. In outpatient samples, six individual isolates were detected which were intermediate/resistant to MER (one in 2009, 2011, 2014 and 2016, and two isolates in 2017; representing 2 *E. coli* and 4 *K. pneumoniae* isolates): one MER-intermediate isolate was resistant to all indicator antibiotics (but susceptible to colistin), while 5 out of 6 were susceptible to GEN and FOS, and 4 out of 6 were susceptible to SXT. In inpatient samples, eight such isolates were detected (one in 2011 and 2013, and two isolates in 2015, 2016 and 2017, respectively; representing 3 *E. coli* and 5 *K. pneumoniae* isolates). All relevant isolates were susceptible to GEN, 6 out of 8 were susceptible to FOS, while 4 out of 8 were susceptible to SXT. Most isolates intermediate/resistant to carbapenems were also resistant to CIP (in both patient groups).

## 4. Discussion

*E. coli* and *Klebsiella* species are the most common cause of urinary tract infections (UTIs) in both community and healthcare settings [1,3,4,6,9,14,47,48,49,50,51,52]. This was further proven in the context of our study, as *E. coli* was isolated in ~46–66%, and *Klebsiella* spp. was isolated in ~3–16% of cases. Their relatively lower prevalence in inpatient isolates is attributable to the more pronounced diversity in the causative agents of nosocomial UTIs, however, their clinical relevance should not be disregarded in either settings. In line with international literature, there was a predominance of females and patients over 50 years of age, among the affected population [1,3,4,6,9,14,47,48,49,50,51,52].

Regarding the local resistance levels, the results of the 10-year survey showed that there has been a pronounced increase in the resistance rates of several antibiotics by the second half of the study period (2013–2017); in contrast, resistance levels of NIT in *E. coli* decreased. There was no single underlying event found that may be responsible for this local advantageous change in NIT resistance levels, although due to the extended and favoured use of fluoroquinolones and the inability to access NIT—both at the Albert Szent-Györgyi Clinical Center and in the country in the recent years—may have had a notable role. Henceforth, the role of NIT may have a renaissance in the therapy of uncomplicated UTIs [61,62]. The comparison of our results with the surveillance data of the European Antimicrobial Resistance Surveillance Network (EARS-Net, [63]) for the relevant time periods is presented in Table 3.

Some of the most concerning developments is the resistance in *Klebsiella* species to 3rd generation cephalosporins (exemplified by ceftriaxone in this survey), where even the lowest levels of resistance were around 15% in the outpatient group and close to 40% in the inpatient group. ESBL production has been observed in large percentage of urinary isolates in this study, especially in *Klebsiella* species. Some difference could be observed in the ratio of ESBL-positive and CRO-resistant strains (0.42–9.90% for *E. coli* and 0–10.25% for *Klebsiella* spp., respectively); this may be due to the fact that CRO resistance may also occur due to ESBL-independent mechanisms, such as AmpCs or other β-lactamases, overexpression of efflux pumps, changes in membrane permeability or porin mutations [64,65,66]. As ESBLs were the only one with public health/infection control significance in our hospital, the exact mechanism of resistance in this minor group of CRO-resistant isolates was not characterized further [34]. There was a significant difference in the rate of ESBL-positivity in *E. coli* among inpatient and outpatient isolates, however, no such different was observed for *Klebsiella*. ESBL-producing isolates have first been detected in nosocomial settings, nevertheless, after the 2000s, this demarcation line exist less and less [29,36]. In Hungary (and specifically in the southern region of the country), the *bla*_CTX-M_ group is the most prevalent, which is associated with carrying resistance determinants to quinolones and aminoglycosides in addition to the relevant β-lactam antibiotics [67]. UTIs caused by these strains cannot be treated with the “conventional” β-lactam antibiotics (penicillin, cephalosporins), but carbapenems remain as a part of the antibiotic armamentarium [68,69]. However, the overuse of these drugs will inevitably lead to selection pressure (the prevalence of carbapenemase-producing *Enterobacteriaceae* (or CRE) is steadily increasing worldwide). Hungary is currently known to be a low-prevalence country for CRE (most of the isolates carry VIM- or OXA-48-like enzymes), however, most of the isolates originate from UTIs [70,71].

Similarly, high levels of resistance to fluoroquinolone antibiotics have been reported for *Enterobacteriaceae* in regions where no restrictions were introduced to their use in the community and they are still considered to be first-line agents. If the Hungarian antibiotic utilization trends are considered, the country is doing well quantitatively (i.e., the amount of antibiotics consumed), however, a qualitative analysis (i.e., targeted therapy) reveals a much bleaker picture: the use of broad-spectrum antimicrobials (including fluoroquinolones) is significantly higher than in Scandinavian countries, which may also correspond to the development of local resistance trends (based on the data of a Hungarian study group and European Surveillance of Antimicrobial Consumption Network; ESAC-Net) [59,60,72,73]. Some novel antibiotics, that recently received marketing authorization (for example, delafloxacin, ceftolozane-tazobactam, ceftazidime-avibactam) may aid the therapy of resistant Gram-negative infections; nevertheless, due to their prohibitive price and the limited clinical experience associated with these drugs, it is unknown when will they be considered in routine therapeutic protocols [42,74,75]. The increase in resistance is not only forcing changes in treatment guidelines and issues in the clinic, but also to poor prognoses, decreased QoL and an increase in the mortality rate of patients, especially in nosocomial settings [5]. *E. coli* and *K. pneumoniae* are major pathogens of nosocomial infections, including UTIs, that is frequently associated with resistance to the critically important antimicrobials. The WHO has recently published a priority list of resistant pathogens, where *Escherichia coli* and *Klebsiella* species resistant to third generation cephalosporins and carbapenems represent top-priority [76].

Some limitations of this study must be acknowledged: firstly, the design of the study is retrospective and there has been an inability to access the medical records of the individual patients affected by these infections. For this reason, the correlation between the existence of relevant risk factors and underlying illnesses (apart from age and inpatient/outpatient status) and the *E. coli/Klebsiella* spp. UTIs could not be assessed. The age-associated incidence in isolation of these pathogens may also reflect (at least in part) the high rate of bacteriuria in the elderly population (especially in catheterized patients) [77]. There is a risk of selection bias, as studies describing the prevalence of infectious diseases and resistance trends are mainly tertiary-care centers, which generally correspond to patients with more severe conditions or underlying illnesses. Finally, the molecular characterization of the resistance determinants in the individual isolates was not performed, only to the extent of presence/absence of ESBLs [78,79].

## 5. Conclusions

This study presents the epidemiological trends and resistance levels of *E. coli* and *Klebsiella* species, the main pathogens associated with urinary tract infections (UTIs) in Hungary, over a long surveillance period (10 years), mainly demonstrating an increasing tendency regarding the resistance levels to various antibiotics. To the best of our knowledge, this is the longest-spanning study reporting on the prevalence and susceptibility patterns of these pathogens (and UTIs caused by these uropathogens by proxy) in Hungary. Their significantly higher prevalence in female patients with advanced age and instrumentation/catheterization and is in line with the findings in the literature. The type of setting (inpatient/outpatient) also had an effect on their isolation frequency, as there was a predominance of *E. coli* in uncomplicated UTIs, while in the inpatient setting, more relevant pathogens may be implicated.

In addition to patient history, drug allergies and national/institutional drug availability, the choice of empiric antibiotic therapy should be selected based on local susceptibility profiles or a cumulative hospital antibiogram, based on the guidelines of the Infectious Diseases Society of America (IDSA); nonetheless, the choice of antimicrobial drugs should be revised after the specific antibiogram for the relevant urinary pathogen has become available. The data in this study may aid the creation of a local/hospital antibiogram or a national surveillance system for urinary tract pathogens in Hungary.

## Figures and Tables

**Table 1:** Percentage of resistant *E. coli* strains to indicator antibiotics from inpatient and outpatient departments (2008–2017).

		2008	2009	2010	2011	2012	2013	2014	2015	2016	2017	Statistics
**CIP R%**	*Outpatient*	*13.28*	16.12	21.53	21.80	19.13	21.64	21.31	25.57	25.69	**25.95**	*p* = 0.0003
	*Inpatient*	*20.19*	23.62	26.85	33.42	37.28	38.41	34.73	**40.60**	40.41	33.25	
**NIT R%**	*Outpatient*	4.99	7.33	**12.37**	9.00	6.06	3.05	3.59	3.12	2.42	*1.03*	n.s. (*p* = 0.264)
	*Inpatient*	7.98	12.39	**20.81**	17.57	7.36	3.34	5.04	2.76	2.05	*1.39*	
**GEN R%**	*Outpatient*	*3.17*	5.77	6.35	7.51	5.58	4.54	6.37	**7.76**	5.16	6.88	*p* < 0.0001
	*Inpatient*	*7.82*	8.75	11.68	14.62	16.69	**19.84**	17.01	17.85	18.21	13.10	
**SUM R%**	*Outpatient*	*21.44*	22.16	23.61	25.32	20.87	24.69	24.82	26.68	24.88	**28.08**	n.s. (*p* = 0.189)
	*Inpatient*	25.82	22.45	21.34	25.55	*11.62*	35.29	34.83	36.24	**39.08**	28.21	
**CRO R%**	*Outpatient*	8.99	7.74	7.55	7.33	*5.58*	6.46	8.57	9.56	8.85	**9.70**	*p* = 0.0003
	*Inpatient*	*6.10*	12.39	16.24	18.55	20.48	26.99	**40.94**	25.19	30.41	20.03	
**ESBL%**	*Outpatient*				6.31	*4.33*	5.50	6.94	**9.15**	7.32	9.02	*p* = 0.0028
	*Inpatient*				8.85	10.59	25.72	**38.97**	23.06	29.08	18.39	

Values in italics represent the lowest resistance levels, boldface (peak) values correspond to the highest resistance levels in the study period; n.s.: not significant.

**Table 2:** Percentage of resistant *Klebsiella* spp. strains to indicator antibiotics from inpatient and outpatient departments (2008–2017).

		2008	2009	2010	2011	2012	2013	2014	2015	2016	2017	Statistics
**CIP R%**	*Outpatient*	37.31	41.48	**45.16**	35.47	40.77	40.38	33.03	38.78	*31.60*	34.08	n.s. (*p* = 0.641)
	*Inpatient*	*23.40*	30.00	35.10	37.50	34.28	35.76	**48.40**	45.57	38.18	37.45	
**GEN R%**	*Outpatient*	27.69	**34.09**	25.81	24.53	27.69	27.40	21.10	28.14	*18.80*	19.10	*p* = 0.0002
	*Inpatient*	**26.24**	13.33	14.42	*13.31*	16.96	15.41	17.99	16.14	16.36	13.62	
**SUM R%**	*Outpatient*	*25.38*	**39.77**	33.33	35.00	33.08	35.58	37.61	39.16	32.80	37.45	n.s. (*p* = 0.399)
	*Inpatient*	27.66	*21.67*	34.62	30.24	23.43	29.07	43.15	**43.99**	36.06	35.74	
**CRO R%**	*Outpatient*	*23.85*	31.25	**37.63**	26.74	36.15	35.10	27.52	35.36	27.20	25.09	*p* = 0.132
	*Inpatient*	23.40	21.11	17.31	20.56	23.67	19.77	41.98	28.48	38.18	25.79	
**ESBL%**	*Outpatient*				26.74	30.00	31.73	25.69	**34.22**	26.00	*23.22*	*p* = 0.243
	*Inpatient*				*10.89*	13.43	18.60	33.53	28.48	**36.06**	23.36	

Values in italics represent the lowest resistance levels, boldface (peak) values correspond to the highest resistance levels in the study period; n.s.: not significant.

**Table 3:** Comparison of the resistance data obtained in this study with the surveillance data of EARS-Net.

		Local Resistance Data (2008–2012)	EARS-Net Surveillance Data for Hungary; 2012 [63]	Local Resistance Data (2013–2017)	EARS-Net Surveillance Data for Hungary; 2017 [63]
*3rd generation cephalosporins*	*E. coli*	5.58–26.99%	**17.40%**	6.46–40.94%	**20.10%**
	*Klebsiella* spp.	*17.31*–*37.63%*	**43.00%**	*19.77*–*35.36%*	**41.10%**
*Fluoroquinolones*	*E. coli*	*13.28*–*21.80*%	**28.90%**	*21.31*–*25.95%*	**30.60%**
	*Klebsiella* spp.	23.40–45.16%	**41.60%**	31.60–48.40%	**41.45%**
*Aminoglycosides*	*E. coli*	3.17–16.69%	**15.10%**	4.54–19.84%	**17.10%**
	*Klebsiella* spp.	*13.31*–*34.09%*	**37.80%**	*18.80*–*27.40%*	**41.50%**
*Carbapenems*	*E. coli*	<0.05%	**<0.05%**	<0.05%	**0.10%**
	*Klebsiella* spp.	<0.05%	**0.30%**	<0.05%	**0.30%**

Values in italics represent resistance levels that were lower than the national average.

## Abbreviations

AST: antimicrobial susceptibility testing; CFU: colony-forming unit; CRE: carbapenem-resistant *Enterobacteriaceae*; CIP: ciprofloxacin; CRO: ceftriaxone; EARS-Net: European Antimicrobial Resistance Surveillance Network; ESBL: extended-spectrum β-lactamase; ESAC-Net: European Surveillance of Antimicrobial Consumption Network; EUCAST: European Committee for Antimicrobial Susceptibility Testing; FOS: fosfomycin; GEN: gentamicin; GP: general practitioner; ID: identification; IDSA: Infectious Disease Society of America; IP: inpatient; LIS: laboratory information system; LPS: lipopolysaccharide; MALDI-TOF MS: matrix-assisted laser desorption/ionization time-of-flight mass spectrometry; MDR: multidrug-resistance; MER: meropenem; NEAK: Hungarian National Health Insurance Fund; NIT: nitrofurantoin; OP: outpatient; OPAT: outpatient parenteral antibiotic therapy; PAI: pathogenicity island; QoL: quality-of-life; SXT: sulfamethoxazole-trimethoprim; UPEC: uropathogenic *Escherichia coli*; UTI: urinary tract infection; WHO: World Health Organization.
